# Psychometric Properties of the Clinical Dementia Rating Scale Sum of Boxes in Parkinson’s Disease

**DOI:** 10.3233/JPD-202390

**Published:** 2021-04-13

**Authors:** Julia Gallagher, Jacqueline Rick, Sharon X. Xie, Pablo Martinez-Martin, Eugenia Mamikonyan, Alice Chen-Plotkin, Nabila Dahodwala, James Morley, John E. Duda, John Q. Trojanowski, Andrew Siderowf, Daniel Weintraub

**Affiliations:** a Department of Neurology, Perelman School of Medicine at the University of Pennsylvania, Philadelphia, PA, USA; b Department of Biostatistics, Epidemiology and Informatics, Perelman School of Medicine at the University of Pennsylvania, Philadelphia, PA, USA; c Center for Networked Biomedical Research in Neurodegenerative Diseases (CIBERNED), Carlos III Institute of Health, Madrid, Spain; d Department of Psychiatry, Perelman School of Medicine, University of Pennsylvania, Philadelphia, PA, USA; e Parkinson’s Disease Research, Education and Clinical Center (PADRECC), Philadelphia Veteran’s Affairs Medical Center, Philadelphia, PA, USA; f Department of Pathology and Laboratory Medicine, Perelman School of Medicine at the University of Pennsylvania, Philadelphia, PA, USA

**Keywords:** Cognition, dementia, Parkinson’s disease, rating scale

## Abstract

**Background:**

A composite measure that assesses both cognitive and functional abilities in Parkinson’s disease (PD) would be useful for diagnosing mild cognitive impairment (MCI) and PD dementia (PDD) and as an outcome measure in randomized controlled trials. The Clinical Dementia Rating Scale Sum of Boxes (CDR-SOB) was designed to assess both cognition and basic-instrumental activities of daily living in Alzheimer’s disease but has not yet been validated in PD.

**Objective:**

To validate the CDR-SOB as a composite cognitive-functional measure for PD patients, as well as to assess its sensitivity to change.

**Methods:**

The CDR-SOB and a comprehensive cognitive and functional battery was administered to 101 PD patients at baseline (39 normal cognition [NC], 41 MCI and 21 PDD by expert consensus panel), and re-administered to 64 patients after 1-2 years follow-up (32 NC and 32 cognitive impairment [CI] at baseline).

**Results:**

Cross-sectionally, CDR-SOB and domain scores were correlated with corresponding neuropsychological or functional measures and were significantly different between cognitive subgroups both at baseline and at follow-up. In addition, CDR-SOB ROC curves distinguished between normal cognition and dementia with high sensitivity, but did not distinguish well between NC and MCI. Longitudinal changes in the CDR-SOB and domain scores were not significant and were inconsistent in predicting change in commonly-used cognitive and functional tests.

**Conclusion:**

The CDR-SOB detects dementia-level cognitive impairment in PD but may not be appropriate for predicting longitudinal combined cognitive-functional changes in patients without significant cognitive impairment at baseline.

## INTRODUCTION

Cognitive impairment, including mild cognitive impairment (MCI) and dementia, are increasingly recognized as common and sometimes debilitating symptoms in Parkinson’s disease (PD). Up to 80% of PD patients will become demented during the course of their disease [1, 2], and patients with established PD and normal cognition who develop MCI subsequently progress to dementia frequently [3].

Properly diagnosing MCI and dementia in PD patients is essential for clinical management, caregiver support, and clinical trial recruitment. In 2007 a Movement Disorder Society (MDS) Task Force published clinical diagnostic criteria for PD dementia (PDD), which include impairment in multiple cognitive domains plus clinically significant functional impairment independent of motor symptoms [4]. In 2012 another MDS Task Force published criteria for PD-MCI, which include cognitive deficits that are not sufficient to interfere significantly with functional independence [5]. While both definitions require or recommend assessment of cognitive abilities across multiple domains and an evaluation of functional abilities, there is no agreed upon gold standard for either, and no single instrument validated to assess both in PD.

There are several PD-specific cognition-related fu-nctional questionnaires. The Penn Parkinson’s Daily Activities Questionnaire (PDAQ-15) is a brief 15-item instrument that assesses cognitive instrumental activities of daily living (iADLs) [6]. A similar instrument is the Parkinson’s Disease Cognitive Functional Rating Scale [7]. In addition, there are performance-based cognitive function instruments applied in PD, including the UCSD Performance-Based Skills Assessment [8] and the Direct Assessment of Functional Status [9, 10]. However, none of these inst-ruments assess cognition and function together. The potential advantage to having such a composite measure is to maximize efficiency in diagnosing PD-MCI and PDD, and to have a combined cognition-function instrument to use as an outcome measure in randomized controlled trials.

The Clinical Dementia Rating Scale Sum of Boxes (CDR-SOB) is a composite measure that was designed to assess both cognition and function in Alzheimer’s disease (AD) [11]. The instrument utilizes a semi-structured interview with both the patient and a care partner, in combination with a series of cognitive tasks to assess performance in three cognitive (memory, orientation, and judgment and problem solving) and three functional (community affairs, home and hobbies, and personal care) domains. The six domain scores are each rated on a 4-point scale from 0 to 3, and a CDR-SOB is generated (range 0–18). Previous research has utilized the CDR-SOB to stage dementia severity in AD [12].

To date there is only one study reporting on CDR-SOB performance in PD[13], which found that the CDR-SOB was better than the CDR-Global Score in characterizing PD-MCI, but the instrument did not distinguish well between those with and without dementia. There are several other studies that have utilized the CDR-SOB as a measure of global cognition in PD [14, 15] or included it in a battery of cognitive tests in PD patients [16, 17], but did not specifically test its psychometric properties.

The current study prospectively administered and evaluated the CDR-SOB in a well-characterized sample of PD patients with established disease, varying levels of cognitive abilities, and consensus process-generated cognitive diagnoses (normal cognition, MCI and dementia). This study also aimed to compare the CDR-SOB to a detailed neuropsychological battery and other instruments that assess ADLs. Additionally, this study examined the CDR-SOB’s sensitivity to change over a 1-2 year follow up period.

## MATERIALS AND METHODS

### Participants

One hundred one patients with idiopathic PD, diagnosed by a movement disorders specialist based on UK Brain Bank criteria [18], and their care partners were recruited from an active clinical research cohort at the Parkinson’s Disease and Movement Disorders Center at the University of Pennsylvania. The clinical cohort is followed longitudinally with annual or biennial assessments of cognition and function. All participants provided written informed consent prior to participation.

### Assessments

#### Clinical

Motor disease severity was measured with the Unified Parkinson Daily Rating Scale (UPDRS) Part III [19], and depression severity with the 15-item Geriatric Depression Scale (GDS-15) [20]. Levodopa equivalence daily dose (LEDD) was calculated [21], and sex, education, disease duration and age were recorded.

#### CDR-SOB

The CDR-SOB was administered either in person (preferred) or over the phone (when necessary) close in time to the participant’s scheduled research visit. The care partner was interviewed first and was administered sections 1–6 of the CDR-SOB. The patient was interviewed second with sections 1–3. Both CDR-SOB and six CDR domain scores were generated, with higher scores indicating worse cognitive performance. The test was jointly scored (by DW and either JR, JG or EM) blind to the patient’s neuropsychological test results and consensus cognitive diagnosis.

#### Neuropsychological assessments

A battery of cognitive tests is administered either annually (up to year 4 of study participation) or biennially (after year 4) to all members of the cohort by trained research staff. The battery includes the Mattis Dementia Rating Scale-2 (DRS-2) and Montreal Cognitive Assessment (MoCA) to measure global cognition, Hopkins Verbal Learning Test (HVLT) to measure memory, Verbal Fluency (FAS), Letter-Number Sequencing (LNS), and Trails B to measure executive function, Symbol Digit Test and Trails A to measure attention, Benton Judgment of Line Orientation (JOLO) and Clock Drawing Test to measure visuospatial function, and Boston Naming Test (BNT), and Verbal Fluency (animals) to measure language. The full neuropsychological battery has been described previously [3, 22].

#### Functional assessments

PD participants and their care partners were also administered the Penn Parkinson’s Daily Activities Questionnaire (PDAQ-15) [6] to assess cognition-related functional abilities and the Alzheimer’s Disease Cooperative Study Activities of Daily Living Inventory (ADCS-ADLi) [23] to assess basic and instrumental functional abilities.

#### Consensus cognitive diagnosis

Assignment of a cognitive consensus diagnosis (normal cognition [NC], MCI, or PDD) was made by a team of physician (movement disorders neurologists and psychiatrist) specialists. All neuropsychological and functional data were considered, and the MCI and PDD diagnostic criteria proposed by the MDS Task Force were applied, in a process described previously [3].

#### Longitudinal methods

Sixty-four participants were re-evaluated one (N = 21) to two (N = 43) years post-baseline with the same assessments. Due to small sample sizes, participants diagnosed with MCI (N = 27) or PDD (N = 5) at baseline were combined into a “cognitive impairment” (CI) group, for comparison with the NC group (N = 32). Consensus diagnosis information was available for 80% (51/64) of participants at follow-up.

### Statistical analyses

To characterize the sample descriptive statistics (mean, standard deviation, median, range, proportion) were used. Acceptability of the CDR-SOB was determined by the distribution of scores, floor and ceiling effects. The criteria for these parameters were: arbitrary limit, 10% of the maximum possible score for the difference between mean and median; maximum acceptable for floor and ceiling effect, 15%; and skewness between –1 and+1. CDR-SOB internal consistency was analyzed by inter-item correlation, item homogeneity coefficient, corrected item-to-total correlation and Cronbach’s alpha. The corresponding standard values were: 0.20–0.75; ≥0.20; ≥0.40; and ≥0.70 [24]. The association between CDR-SOB scores and other measures in the study was explored with the Spearman rank correlation coefficient, as most of the variables are ordinal and sho-wed non-normal distribution (Shapiro-Francia test). Coefficient values ≥0.60 were considered high and 0.30–0.59 moderate [25]. CDR-SOB scores were presented by the classification NC, MCI, or PDD, and the Kruskal-Wallis test was applied to compare the scores according to these groups, with the Bonferroni correction applied for multiple comparisons. Criterion-based validity was assessed using CDR-SOB cut-off points to distinguish consensus cognitive state from each other by means of ROC analysis. Analyses were conducted with SPSS (IBM SPSS Statistics for Windows, Version 23.0. Armonk, NY: IBM Corp.) and Stata 15.1 (Stata Corporation, College Station, TX, USA).

For longitudinal analyses, CDR-SOB scores were organized according to a diagnosis of NC or CI, and the Wilcoxon-Mann-Whitney test was applied to compare these groups at each time point. The Chi-square test was utilized to compare sex between groups at each time point. Linear mixed-effects models were used to examine the change in CDR-SOB and CDR domains over time, both for the entire cohort and by cognitive subgroup. Similar models were utilized to examine progression over time on commonly-used cognitive and functional measures (i.e., DRS-2, MoCA, PDAQ-15 and ADLI) and to determine association between longitudinal changes in CDR-SOB score and these measures. All statistical tests were two-sided, and statistical significance was set at 0.05 level.

## RESULTS

### Subject characteristics

Table 1 lists the subject demographic and clinical characteristics. Of the 101 participants, 39 were diagnosed NC, 41 MCI, and 21 PDD at their most recent cognitive consensus conference. The PDD group was older (*p* = 0.02 and had higher UPDRS III scores *p* < 0.001) than the NC group. In addition, the GDS-15 score was significantly higher in the MCI group compared to the NC group (*p* = 0.005). Percentage male, education level, LEDD values and disease duration were not significantly different between the groups (all p values > 0.05). As expected, progression from NC to MCI to PDD correlated with worsening performance on cognitive measures (all p values < 0.05). This pattern was also observed in assessments of functional abilities (Table 1).

**Table 1:** Baseline clinical and demographic characteristics

Variable	Cross-sectional cohort						Longitudinal cohort				
	N	Total cohort median (IQR)	NC median (IQR)	MCI median (IQR)	PDD median (IQR)	Kruskal-Wallis	N	Total cohort median (IQR)	Normal Cognition median (IQR)	Cognitive Impairment median (IQR)	Wilcoxon-Mann-Whitney
		(N = 101)	(N = 39)	(N = 41)	(N = 21)	*p* value		(N = 64)	(N = 32)	(N = 32)	*p* value
Education (years)	101	16 (14–18)	18 (16–18)	16 (14–18)	16 (14–18)	0.26	64	16 (14–18)	18 (16–18)	16 (14–18)	0.10
Sex (% male)	101	66.3	53.8	78	66.7	0.07	64	70.3	56.3	84.4	0.01
Disease duration	101	9 (6–13)	9 (6–11)	10 (6–14)	11 (7–18)	0.15	64	14 (10–19)	13 (10–19)	16 (10–18)	0.31
(years)
GDS-15	99	2 (1–4)	1 (0–2)	3 (2–5)	3 (2–4)	<0.001	64	2 (1–4)	1 (0.25–2)	3 (2–5)	0.003
UPDRS III	100	30 (23–41)	23 (17–32)	33 (24–46)	42 (32–51)	<0.001	64	28 (20–40)	23 (18–32)	34 (24–44)	0.003
LEDD	101	600 (400–1025)	550 (350–913)	641 (450–1332)	800 (400–1030)	0.21	64	575 (400–973)	480 (300–922)	600 (400–1000)	0.22
Age at test	101	72 (67–76)	70 (64–75)	72 (69–76)	76 (72–80)	0.01	64	71 (64–75)	70 (63–72)	72 (71–77)	0.01
(years)
DRS-2	101	135 (129–140)	140 (139–141)	134 (131–137)	121 (112–128)	<0.001	64	138 (133–141)	140 (139–141)	134 (130–137)	<0.001
MoCA	101	24 (21–27)	27 (25–28)	23 (22–25)	17 (16–21)	<0.001	64	25 (23–27)	27 (26–29)	23.0 (20–24)	<0.001
Clock	87	6 (5–7)	7 (6-7)	5 (4–6)	4 (3–6)	<0.001	61	5 (3–8)	5 (3–8)	7 (3–9)	0.51
Drawing Test
Boston	69	58 (56–59)	59 (57–60)	58 (55–59)	56 (26–59)	0.04	52	59 (57–59)	59 (59-60)	56 (53–59)	<0.001
Naming Test
JOLO	83	24 (20–26)	24 (22–26)	24 (20–26)	16 (13–18)	0.001	59	24 (20–26)	25 (23–28)	22 (20–26)	0.02
Trails A	80	45 (35–70)	38 (31–45)	60 (40–81)	102 (77–113)	<0.001	58	43 (32–57)	35 (28–44)	47 (39–67)	0.002
Trails B	79	97 (77–154)	79 (61–93)	131 (96–209)	272 (167–300)	<0.001	57	88 (73–129)	79 (59–88)	122 (95–180)	<0.001
Symbol Digit	90	31 (20–40)	40 (35–45)	23 (19–31)	13 (8–15)	<0.001	61	35 (23–41)	41 (35–45)	29 (22–35)	<0.001
Modalities Test
LNS	88	9 (7–11)	10 (9–12)	8 (6–9)	5 (4–7)	<0.001	64	9 (7–11)	10 (9–12)	8 (6–9)	0.002
HVLT immediate	90	21 (15–26)	25 (21–29)	19 (14–24)	13 (7–20)	<0.001	64	23 (16–27)	27 (24–29)	17 (14–23)	<0.001
recall
HVLT delayed	90	7 (3–9)	8 (6–11)	5 (2–8)	0 (0–7)	<0.001	64	7 (5–9)	9 (7–11)	5 (4–7)	<0.001
recall
HVLT recognition	90	10 (8–11)	11 (10–12)	9 (8–10)	8 (5–10)	<0.001	64	10 (9–11)	11 (10–12)	10 (8–11)	0.012
discrimination
FAS fluency	90	41 (30–51)	47 (40–58)	36 (26–44)	27 (19–35)	<0.001	64	41 (35–50)	44 (39–62)	36 (25–44)	0.002
Animal fluency	90	16 (12–20)	20 (16–23)	15 (11–17)	9 (7–12)	<0.001	64	17 (13–22)	21 (18–23)	15 (12–18)	<0.001
ADCS-ADLi	98	73 (64–77)	75 (73–78)	70 (64–77)	60 (45–65)	<0.001	63	74 (69–77)	75 (72–77)	72 (59–77)	0.11
PDAQ-15	99	49 (38–55)	55 (50–59)	48 (40–54)	27 (20–39)	<0.001	64	53 (44–57)	55 (50–58)	48 (35–57)	0.008

### CDR characteristics

Table 2 lists the CDR-SOB and domain scores for the total cohort and by cognitive subgroup. Cron-bach’s alpha coefficient was 0.94; inter-item correla-tion ranged from 0.46 (orientation-personal care) to 0.87 (hobbies-community), with an item homogeneity coefficient of 0.64; and item-total correlation from 0.75–0.89. The scores that were significan-tly higher (i.e., worse) across the three groups (PDD > MCI>NC) were Memory (H(2)=46.86, *p* < 0.001), Judgment (H(2)=57.10, *p* < 0.001), Community (H(2)=47.64, *p* < 0.001) and Hobbies (H(2) = 54.18, *p* < 0.001) domains, and CDR-SOB (H(2)=59.58, *p* < 0.001). In addition, the PDD group scored significantly higher than the NC group in the Orientation (H(2)=38.134, *p* < 0.001) and Personal Care domains (H(2)=35.98, *p* < 0.001). There was a high floor effect for all CDR domains, but no ceiling effect. Controlling for age, there was a weak correlation between CDR-SOB and UPDRS III scores at baseline (*r* = 0.27; *p* = 0.008).

**Table 2:** CDR-SOB and CDR domain scores at baseline

CDR	Cross-sectional cohort								Longitudinal cohort			
	Cohort median (IQR)	Min	Max	Floor effect^*^	NC median (IQR)	MCI median (IQR)	PDD median (IQR)	Kruskal-Wallis	Cohort mean (SD)	NC mean (SD)	CI mean (SD)	Wilcoxon-Mann-Whitney
								*p* value^¶^				*p* value
SOB	1.5 (0.5–3.5)	0	13	15.8	0.5 (0–1.5)	2 (1–3)	6 (3.8–9.5)	<0.001^∥^	1 (0.5–2.5)	0.5 (0–1.4)	2 (1–5)	0.001
Memory	0.5 (0–1)	0	3	27.7	0 (0–0.5)	0.5 (0.5–1)	1 (1–1.5)	<0.001^∥^	0.5 (0–0.5)	0.3 (0–0.5)	0.5 (0.5–1)	<0.001
Orientation	0 (0–0.5)	0	2	50.5	0 (0–0.5)	0 (0–0.5)	1 (0.5–1.5)	<0.001^‡^§	0 (0–0.5)	0 (0–0.5)	0 (0–0.5)	0.39
Judgment	0.5 (0.5–1)	0	2	22.8	0 (0–0.5)	0.5 (0.5–1)	1 (1–2)	<0.001^∥^	0.5 (0–0.5)	0.3 (0–0.5)	0.5 (0.5–1)	<0.001
Community	0 (0–0.5)	0	2	57.4	0 (0–0)	0 (0–0.5)	1 (0.5–1.5)	<0.001^∥^	0 (0–0.5)	0 (0–0)	0 (0–1)	0.03
Hobbies	0 (0–0.5)	0	3	55.5	0 (0–0)	0 (0–0.5)	1 (0.8–2)	<0.001^∥^	0 (0–0.5)	0 (0–0)	0.5 (0–1)	0.001
Personal Care	0 (0–0)	0	2	78.2	0 (0–0)	0 (0–0)	1 (0–2)	<0.001^‡^§	0 (0–0)	0 (0–0)	0 (0–1)	0.009

¶Bonferroni correction applied to account for multiple comparison across groups. ^*^Floor effect represents the percentage of the total cohort with a value of 0 for the domain score. ^†^NC significantly different than MCI; ^‡^NC significantly different than PDD; §MCI significantly different than PDD; ∥All groups significantly different from one another.

### Correlations with other measures

The CDR Memory domain was moderately correlated with all HVLT subscores (immediate recall *r* = –0.50, delayed recall *r* = –0.53 and recognition recall *r* = –0.43), and CDR Judgment scores were moderately correlated with the three executive tasks in our battery (LNS *r* = –0.45, FAS *r* = –0.49 and Trails B *r* = –0.43). In addition, the CDR functional measures (Community, Hobbies and Personal Care) had moderate to strong correlations with the ADLi and PDAQ scores (r values from –0.58––0.68). The CDR-SOB was strongly correlated with the two global cognitive measures (DRS-2 *r* = –0.67 and MoCA *r* = –0.68). Table 3 lists the correlations which were statistically significant (p values < 0.05).

**Table 3:** Spearman correlation coefficients for CDR-SOB and domain-specific measures

Variable	CDR-SOB Score					
	Memory	Judgment	Community	Hobbies	Personal Care	Total score
HVLT immediate recall	–0.5^*^
HVLT delayed recall	–0.53^*^
HVLT recognition discrimination	–0.43^*^
LNS		–0.45^*^
FAS fluency		–0.39^*^
Trails B		–0.49^*^
ADCS-ADLi			–0.63^**^	–0.58^*^	–0.59^*^
PDAQ-15			–0.68^**^	–0.67^**^	–0.59^**^
DRS-2						–0.67^**^
MoCA						–0.68^**^

^*^Moderate correlation (*r*≥0.3–0.59). ^**^Strong correlation (*r*≥0.6).

### ROC curves

**Fig. 1:**
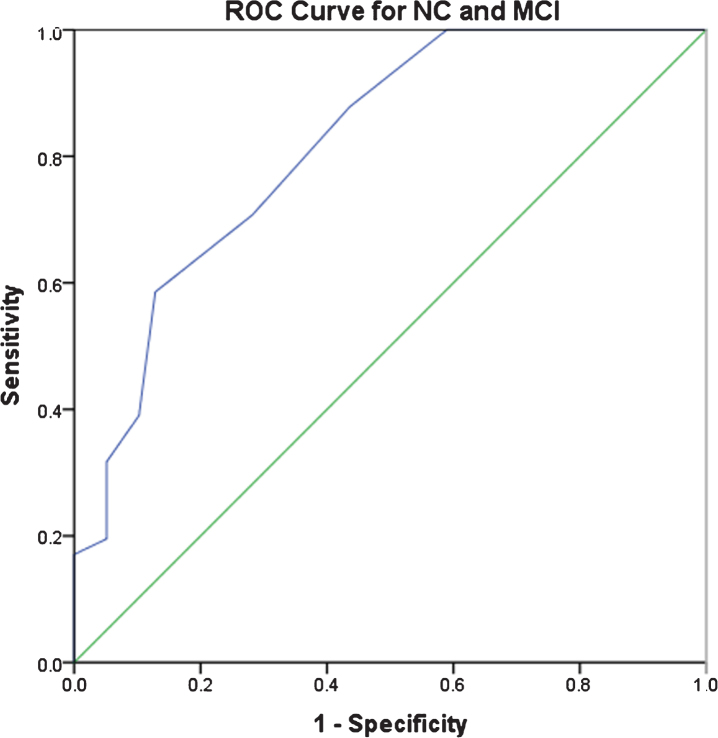
ROC Curves for discriminating NC, MCI, and PDD.

Figure 1 shows the ROC curves for distinguishing NC from MCI and MCI from PDD. For distinguishing MCI from NC, the AUC was 0.82 (95% CI = 0.72–0.91), and the optimal cut-off was a CDR-SOB score of 1.5, with sensitivity and specificity of 0.71 and 0.72. For distinguishing PDD from MCI, the AUC was 0.94 (95% CI = 0.86–0.99), and the optimal CDR-SOB cut-off score was 3.5, with sensitivity and specificity as 0.95 and 0.80. Cognitive instruments (i.e., MoCA and DRS-2) performed as well as or better than the CDR-SOB in distinguishing NC from MCI (MoCA AUC = 0.83, DRS-2 AUC = 0.89) and NC from PDD (MoCA AUC = 0.99, DRS-2 AUC = 1.0).

### Longitudinal results

Sixty-four patients (NC = 32 and CI = 32 at baseline) were re-evaluated (mean [SD] time to follow-up=21.3 [5.8] months) (Tables 1 and 2). Of those that were not reached for follow-up from the cross-sectional sample (N = 38), 7 died, 8 dropped out or were end-pointed, 3 were too sick to participate and 20 were lost to follow-up. The average CDR-SOB score for the CI group was 2.4 points worse than the NC group at baseline, and 3.0 points worse at follow-up (Supplementary Table 1). Annual changes in CDR-SOB and domain scores were not statistically significant either in the entire cohort or by cognitive subgroup (Table 4 and Supplementary Table 2). However, a significant decline was seen in DRS-2, MoCA and PDAQ-15 scores over time (Supplementary Table 3). Change in CDR-SOB predicted changes in the MoCA (*p* = 0.02) and ADLI (*p* = 0.001) scores, but not in the DRS-2 or PDAQ-15 scores (Supplementary Table 4).

**Table 4:** Annual change in CDR-SOB and CDR domain scores

Test	Estimate	Standard Error	t	df	*p* value for annual change
CDR-SOB	0.08	0.11	0.68	63.7	0.50
CDR Memory	0.02	0.03	0.83	64.1	0.41
CDR Orientation	–0.002	0.03	–0.09	64.5	0.93
CDR Judgment	–0.01	0.03	–0.25	64.3	0.81
CDR Community Affairs	–0.001	0.02	–0.03	63.8	0.98
CDR Home &Hobbies	0.02	0.03	0.68	64.2	0.50
CDR Personal Care	0.04	0.03	1.24	64.5	0.22

## DISCUSSION

We assessed the psychometric properties, including discrimination of consensus process-derived cognitive diagnoses, of the CDR-SOB as a combined cognitive-functional assessment tool in PD patients with a range of cognitive abilities. The CDR-SOB has the unique advantage of being an instrument that queries care partners for changes in cognition and ADLs while also directly assessing a patient’s cognitive abilities through an abbreviated neuropsychological battery. Our cohort had a mix of cognitive diagnoses that largely reflect what is seen in routine clinical care.

We found that CDR-SOB score and domain specific scores showed statistically significant, although overlapping, differences between NC, MCI and PDD groups, and were significantly correlated with their corresponding neuropsychological or functional measures. Additionally, the sensitivity and specificity of the instrument were high for discriminating dementia from MCI, but suboptimal for discriminating MCI from NC.

The internal consistency of the instrument items was high, yet there was a sizeable floor effect (i.e., toward being intact), particularly for two of the functional domains (home/hobbies, personal care). This suggests that PD patients with NC, MCI and even mild dementia generally have preserved basic ADL function (e.g., bathing, toileting and dressing), even as instrumental ADLs (e.g., handling financial affairs, meal preparation, medication coordination) are impaired. If our cohort had included more patients with dementia, then impairments in these two domains likely would have been observed.

The instrument did not perform as well in distinguishing between NC and MCI, with no cut-off score having both adequate sensitivity and specificity. This in part reflects the floor effect of the instrument, or lack of sensitivity to mild changes, with MCI patients overall showing relatively little impairment on the instrument.

While CDR-SOB scores were statistically different between PD patients with and without CI at a single time point, it may not be sensitive to change over the medium term, at least for patients with relatively intact cognitive performance at baseline. Minimal, statistically insignificant annual changes, including by cognitive subgroup, in both CDR-SOB and domain scores, suggests that the scale is insensitive to detect cognitive-functional changes over a 1-2 year period in PD patients with predominantly normal cognition or MCI at baseline. In addition, other commonly-used cognitive and functional measures were sensitive to change over the same time period. Even dividing the sample into cognitive subgroups to enable patients with CI at baseline to be examined separately did not change the results. Although changes in CDR-SOB did correlate with changes in some cognitive and functional measures (i.e., the MoCA and ADLI), this was not consistent (i.e., not the DRS-2 and PDAQ-15), and these disparate findings defy easy explanation. Thus, although the CDR-SOB statistically differentiates between PD cognitive subgroups cross-sectionally, its between-group overlap and inability to predict change, coupled with its inconsistent correlation with related instruments, brings into question its validity as a composite tool for cognitive and functional assessment in PD patients.

There were limitations to the study which will need to be addressed in future research in this area. First, the time lag between when a patient completed the CDR-SOB and when they completed the full research battery was variable and could be up to a year (mean = 104 days at baseline and 83 days at follow up). Second, some patients were not able to complete the entire research battery because of fatigue, time constraints or other reasons. In addition, not all care partners completed the ADCS-ADLi and PDAQ-15. Furthermore, the sample size for the dementia group was significantly smaller than for the other two cognitive groups. Finally, a relatively short follow-up time limited the opportunity for the CDR-SOB to detect actual changes over time in cognition and functional abilities.

This study demonstrated that while the CDR-SOB can be used as a composite cognitive-functional measure to detect cognitive impairment in PD patients, particularly when trying to differentiate dementia from MCI, it may not be suitable for detecting longitudinal changes in PD patients over the medium term, at least in those with more intact cognition. Additional research with longer follow-up times and a larger cohort of patients with cognitive impairment at baseline may be needed to better determine the sensitivity to change of the CDR-SOB in PD.

## CONFLICT OF INTEREST

Drs.Weintraub, Dahodwala and Siderowf received salary support from MJFF for work on the Parkinson’s Progression Markers Initiative.

## Supplementary Material

Supplementary Material
