# Supplementary material for: Psychometric Properties of the Clinical Dementia Rating Scale Sum of Boxes in Parkinson’s Disease
Source: J Parkinsons Dis. 2021 Apr 13;11(2):737–45. doi: 10.3233/JPD-202390 (PMC8058172; doi:10.3233/JPD-202390)
Supplement: Supplementary Material [file jpd-11-jpd202390-s001.pdf]

Supplementary Material

Psychometric Properties of the Clinical Dementia Rating Scale Sum of Boxes in Parkinson's Disease

Supplementary Table 1. Longitudinal cohort CDR-SOB scores by cognitive diagnosis

Group	Baseline					Follow-up				
	N	Mean	Median (IQR)	Range	Wilcoxon-Mann-Whitney p value*	N	Mean	Median (IQR)	Range	Wilcoxon-Mann-Whitney p value*
Total	64	2.0 (2.5)	1 (0.5-2.5)	13		51	2.4 (3.7)	1 (0.5-2.5)	17	
Normal Cognition	32	0.8 (0.9)	0.5 (0-1.4)	3.5	0.001	24	0.8 (0.9)	0.5 (0-1.4)	3.5	<0.001
Cognitive Impairment	32	3.2 (3.0)	2.0 (1-4)	12.5		27	3.8 (4.6)	2.0 (1-4.5)	16.5	

* Comparison between normal cognition and cognitive impairment groups

Supplementary Table 2. Annual change in CDR-SOB and domain scores by cognitive subgroup at baseline

Test	Normal cognition	Cognitive impairment	Difference	Standard Error	t	df	p value for annual change
CDR-SOB	-0.01	0.17	-0.18	0.23	-0.80	62.8	0.43
Memory	0.02	0.02	0.002	0.06	0.04	63.3	0.97
Orientation	-0.04	0.04	-0.08	0.05	-1.42	63.5	0.16
Judgment	0.01	-0.02	0.03	0.05	0.47	63.6	0.64
Community Affairs	-0.01	0.01	-0.02	0.04	-0.42	62.9	0.68
Home & Hobbies	0.01	0.04	-0.03	0.06	-0.54	63.3	0.59
Personal Care	-0.001	0.08	-0.09	0.06	-1.36	63.5	0.18

Supplementary Table 3. Annual change in reference cognitive and functional measures in longitudinal cohort

Test	Estimate	Standard Error	t	df	p value for annual change
DRS-2	-2.26	0.61	-3.68	64.63	<0.001
MoCA	-0.62	0.22	-2.86	64.24	0.006
ADLI	-0.93	0.48	-1.94	62.62	0.06
PDAQ-15	-1.32	0.59	-2.26	63.88	0.03

Supplementary Table 4. Association between change in CDR-SOB and changes in cognitive and functional measures in longitudinal cohort

Test	Estimate	Standard Error	t	df	p value for annual change
DRS-2	-1.25	1.80	-0.69	84.4	0.49
MoCA	-1.43	0.60	-2.37	75.0	0.02
ADLI	-4.60	1.35	-3.41	69.5	0.001
PDAQ-15	1.57	1.91	0.83	72.0	0.41
